# Effect of Gegen Qinlian Decoction on Cardiac Gene Expression in Diabetic Mice

**DOI:** 10.1155/2017/7421761

**Published:** 2017-12-12

**Authors:** Jing Han, Zhenglin Wang, Wei Xing, Yueying Yuan, Yi Zhang, Tiantian Lv, Hongliang Wang, Yonggang Liu, Yan Wu

**Affiliations:** ^1^Institute of Chinese Medicine, Beijing University of Chinese Medicine, Beijing 100029, China; ^2^College of Basic Medicine, Key Laboratory of Ministry of Education (Syndromes and Formulas), Key Laboratory of Beijing (Syndromes and Formulas), Beijing University of Chinese Medicine, Beijing 100029, China; ^3^Modern Research Center for Traditional Chinese Medicine, Beijing University of Chinese Medicine, Beijing 100029, China; ^4^College of Chinese Medicine, Beijing University of Chinese Medicine, Beijing 100029, China

## Abstract

The aim of this research is to investigate the therapeutic effect of GGQL decoction on cardiac dysfunction and elucidate the pharmacological mechanisms. db/db mice were divided into DB group or GGQL group, and WT mice were used as control. All mice were accessed by echocardiography. And the total RNA of LV tissue samples was sequenced, then differential expression genes were analyzed. The RNA-seq results were validated by the results of RT-qPCR of 4 genes identified as differentially expressed. The content of pyruvate and ceramide in myocardial tissue was also measured. The results showed that GGQL decoction could significantly improve the diastolic dysfunction, increase the content of pyruvate, and had the trend to reduce the ceramide content. The results of RNA-seq showed that 2958 genes were differentially expressed when comparing the DB group with the WT group. Among them, compared with the DB group, 26 genes were differentially regulated in the GGQL group. The expression results of 4 genes were consistent with the RNA-seq results. Our study reveals that GGQL decoction has a therapeutic effect on diastolic dysfunction of the left ventricular and the effect may be related to its role in promoting myocardial glycolysis and decreasing the content of ceramide.

## 1. Introduction

There is a dramatically increasing epidemic of DM patients. The prevalence of DM is 4% in 1995, and this number is anticipated to reach 5.4% in 2025, amounting to 300 million DM patients [1]. Diabetic cardiomyopathy (DCM) is a major complication of diabetes, afflicting 12% of patients. The prevalence rate will reach 22% in people aged > 64 years old [2].

A very significant aspect in early period of DCM is left ventricular (LV) dysfunction, especially diastolic dysfunction. And it is characterized by LV hypertrophy and increased cardiac fibrosis [3].

Despite of intensive glycemic, lipidemic control and neurohormonal antagonists, the progress of DCM in diabetic patients has not impeded. Even worse, cardiovascular mortality has increased due to hypoglycemia [4]. In brief, the therapeutic rules for DCM come from the treatment of heart failure and DM. No specific medications for this disease have been put to clinical use. Therefore, more studies are required to explore new agents for this complex syndrome.

Gegen Qinlian (GGQL) decoction, which is composed of Radix Puerariae (ge gen), Radix Scutellariae (huang qin), *Coptis chinensis* Franch (huang lian), and Radix Glycyrrhizae Praeparata (zhi gan cao), has been addressed for its medicinal effect against DM for almost ten years [5]. It has been reported that GGQL decoction has the ability to improve hyperglycemia and hyperlipidemia [6, 7]. In addition, GGQL decoction has a positive inotropic, negative frequency effect on the isolated perfused rat heart [8]. Furthermore, R *Scutellariae baicalensis* reduced myocardial infarct size in myocardial ischemia-reperfusion injured rats [9]. Berberine, a compound from *C. chinensis,* has negative chronotropic, positive inotropic, antiarrhythmic, and vasodilator properties [10]. Thereafter, we speculate that GGQL decoction will have protective effect against DCM.

In recent years, transcriptomics has been used to determine the mechanism of traditional Chinese medicine [11]. Thus, the purpose of this study is to evaluate the effect of GGQL decoction on DCM and elucidate the pharmacological mechanisms by transcriptomics. First, we assessed the efficacy of GGQL decoction against injured cardiac functions using the DM mouse model. Then, we evaluate the influence of GGQL decoction on changes of transcriptomics. Some of the differently expressed genes were confirmed by RT-qPCR. Our extensive studies will determine the potential targets of GGQL decoction and provide new treatment strategies for DCM.

## 2. Materials and Methods

### 2.1. Preparation of Gegen Qinlian Decoction

To prepare the aqueous extract of Gegen Qinlian decoction, firstly, Radix Puerariae, Radix Scutellariae, *Coptis chinensis* Franch, and Radix Glycyrrhizae Praeparata, at the rate of 5 : 3 : 3 : 2, were soaked in 10 times of distilled water (v/w) for 30 minutes and then boiled for 1 h. The decoction was filtered and collected. Secondly, the residue was added into 10 times of distilled water (v/w) and boiled for 1 h, and the hot decoction was filtered. Thirdly, the filtrate was mixed and concentrated to the aqueous extract.

### 2.2. Animals and Grouping

Studies were performed following the Guide for the Care and Use of Laboratory Animals published by the National Institutes of Health and with the permission of the Care Committee of Beijing University of Chinese Medicine. Male C57BL/KsJ db/db mice and their similar genetic background age-matched C57BL/KsJ wild-type (WT) mice were obtained from the Nanjing Biomedical Research Institute of Nanjing University (Jiangsu, China). Mice were kept in the animal house with a 12 : 12 h light-dark cycle and controlled temperature of 22–25°C. At 8 weeks of age, db/db mice were randomly divided into the DB group or the GGQL group, and the age-matched WT mice were used as control. The aqueous extract of GGQL decoction was dissolved in distilled water, and the GGQL group was intragastrically administered at a dosage of 23.4 g crude drugs/kg/d for 8 weeks. The DB group and WT group were treated with an equal volume of distilled water. After 8 weeks' administration, all the mice were weighted and the blood glucose was measured. Then, all the mice were sacrificed, and the hearts were harvested. And the tissue of the left ventricle was collected and stored in the liquid nitrogen for subsequent mRNA isolation.

### 2.3. Echocardiographic Assessment

At the 8th week of administration, the mice were accessed by echocardiography. All the mice were taped on the heated procedure board and anesthetized with 1.5% isoflurane in oxygen. In the apical four-chamber view, the peak velocity of early diastolic mitral inflow velocity (*E*), the peak value of late diastolic mitral inflow velocity (*A*), and the E-to-A ratio (*E*/*A*) were measured by pulsed Doppler mode, using Vevo 2100 Imaging System (VisualSonics, Canada) with a 30 MHz high-frequency transducer. In tissue Doppler mode, the early diastolic velocity (*E*') and the late diastolic velocity (*A*') were measured, and ratio of early to late diastolic velocities (*E*'/*A*') was calculated. Data analysis was performed with the use of Vevo 2100 Analytic Software (VisualSonics, Canada).

### 2.4. RNA Isolation and Quality Control

Total RNA was isolated from 15 LV tissue samples (5 samples each group) using Trizol reagent (Invitrogen, CA). Agarose gel electrophoresis was used to analyze RNA integrity and the presence of DNA contamination of samples. RNA purity (ratio of OD260/280 and OD260/230) was measured by NanoPhotometer spectrophotometer. RNA concentration was accurately quantified by Qubit 2.0 Fluorometer. And RNA integrity was accurately detected by Agilent 2100 bioanalyzer. Then, poly-A tail mRNA was enriched with Oligo(dT) beads, and the enriched mRNA was randomly fragmented by divalent cations in NEB Fragmentation Buffer. The fragmented mRNA was reverse transcripted, and the cDNA was synthesized. After end repair and addition of A tail and adaptor to the purified cDNA, cDNAs were selected about 200 bp with AMPure XP beads, PCR amplification was performed, and then the PCR product was purified. The insert size of library was detected by Agilent 2100 bioanalyzer, and the effective concentration of the library was accurately quantified.

### 2.5. RNA Sequencing

The mRNA was sequenced with Illumina HiSeq2500 platform. The read numbers mapped of each gene were counted with HTSeq v0.6.1. RPKM (reads per kilobase of exon model per million mapped reads) of each gene was calculated on the basis of the gene length and reads count mapped to the gene.

Gene Ontology (GO) and Kyoto Encyclopedia of Genes and Genomes (KEGG) enrichment was applied by the GOseq R package and KOBAS. The results of clustered analysis of differentially expressed genes were showed with the heatmap.

### 2.6. RT-qPCR Validation of RNA-seq

Four genes identified as differentially expressed were randomly selected to validate the results of RNA-seq by RT-qPCR. 1 *μ*g of total RNA was reverse transcribed to cDNA in 20 *μ*l reaction. Custom gene-specific primers for qRT-PCR were designed by Primer-BLAST, and the sequences of primers are listed in Table 1. Gene expression was measured in triplicates using the ABI StepOnePlus® instrument (ABI, USA) and SYBR GreenImaster mix (Roche, USA). The protocol of the reactions was 2 min at 50°C and 10 min at 95°C, followed by 40 cycles of denaturation at 95°C for 15 s and annealing at the corresponding melting temperatures for 30 s, and the melt curve was detected from 60°C to 95°C (0.5°C increments every 5 s). All the expression levels of mRNA were normalized with GAPDH as a housekeeping gene.

### 2.7. Measurement of Ceramide in Myocardial Tissue

On the day of extraction, the myocardial tissues were weighed with an analytical balance. The tissues were grinded, and cold 2: 1 chloroform: methanol was added. After vortexing, the samples were stored overnight at 4°C. After centrifugation, the organic phase was extracted and dried under nitrogen. The samples were reconstituted for UPLC/MS/MS analysis. The content of ceramide was measured with a UPLC/MS/MS. Ceramide were analyzed using an Agilent TQ-S triple quadrupole mass spectrometer with positive ion electrospray ionization (ESI) source by Daughters Scan. The chromatographic separation was performed by a Waters UPLC Acquity. A reverse-phase Acquity BEH C18 column (2.1 × 100 mm, 1.7 *μ*m) was used as the analytical column. Chromatographic separation was carried out in binary gradient with 2 mM ammonium formate and 0.2% formic acid in water as solvent A and 1 mM ammonium formate and 0.2% formic acid in methanol as solvent B, and the flow rate was 0.4 ml/min. The column temperature was 40°C, and the injection volume was 2 *μ*l.

### 2.8. Measurement of Pyruvate in Myocardial Tissue

The myocardial tissues were weighed about 20 mg with an analytical balance. The protein concentration and pyruvate content of each tissue homogenate were measured according to the test kits.

### 2.9. Statistical Analysis

Differential expression analysis between each two groups was measured by using the DESeq R package (1.10.1), which provides statistical routines to determine differential expression in digital gene expression data using a model based on the negative binomial distribution. The *P* values were adjusted with the Benjamini and Hochberg's approach for controlling the false discovery rate (FDR). Genes with an adjusted *P* value < 0.05 were considered to be differentially expressed.

All the data were presented as mean ± SEM. Statistical analysis was performed with one-way analysis of variance (ANOVA) by SPSS program package (version 17.0). It is considered to be statistical significant when *P* value was below 0.05.

## 3. Results

### 3.1. Blood Glucose and Bodyweight

**Figure 1:**
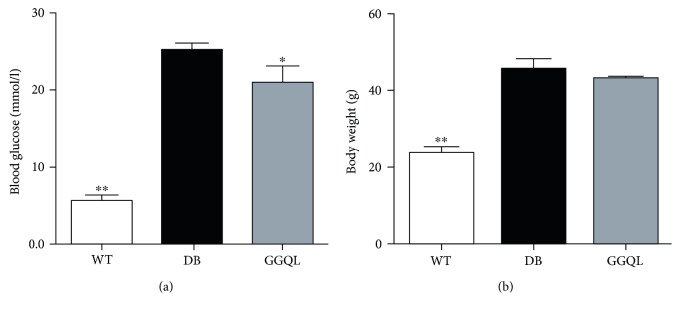
The results of blood glucose and bodyweight. (a) Blood glucose and (b) bodyweight in each group (*n* = 8). The DB group was the reference group to calculate *P* values, ^∗^*P* < 0.05 and ^∗∗^*P* < 0.01.

The results showed that the blood glucose and bodyweight of db/db mice were significantly higher than those of WT mice. Gegen Qinlian decoction could significantly reduce the blood glucose of the diabetic mice, but it had no significant effect on the bodyweight of the diabetic mice (Figure 1).

### 3.2. Effect of GGQL Decoction on Cardiac Function

**Figure 2:**
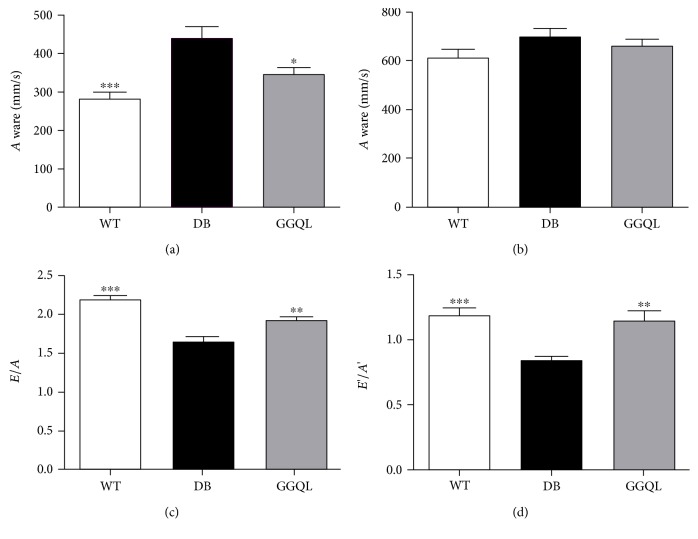
The results of cardiac function detected by echocardiograph. (a) *E* wave, (b) *A* wave, (c) *E*/*A* ratio, and (d) *E*'/*A*' ratio in each group (*n* = 9). The DB group was the reference group to calculate *P* values, ^∗^*P* < 0.05, ^∗∗^*P* < 0.01 and ^∗∗∗^*P* < 0.001.

The results of echocardiography showed that *A* wave of the DB group increased significantly, and *E*/*A* ratio and *E*'/*A*' ratio decreased markedly compared to the WT group. The results indicated that the diastolic function of the left ventricle was impaired. After 8 weeks of treatment, compared to the DB group, *E*/*A* ratio and *E*'/*A*' ratio were recovered, and *A* wave was also downregulated, suggesting that GGQL decoction could improve the diastolic dysfunction caused by diabetes mellitus. In three groups, there was no significant difference in *E* wave (Figure 2).

### 3.3. Gene Expression Levels and DEGs

**Figure 3:**
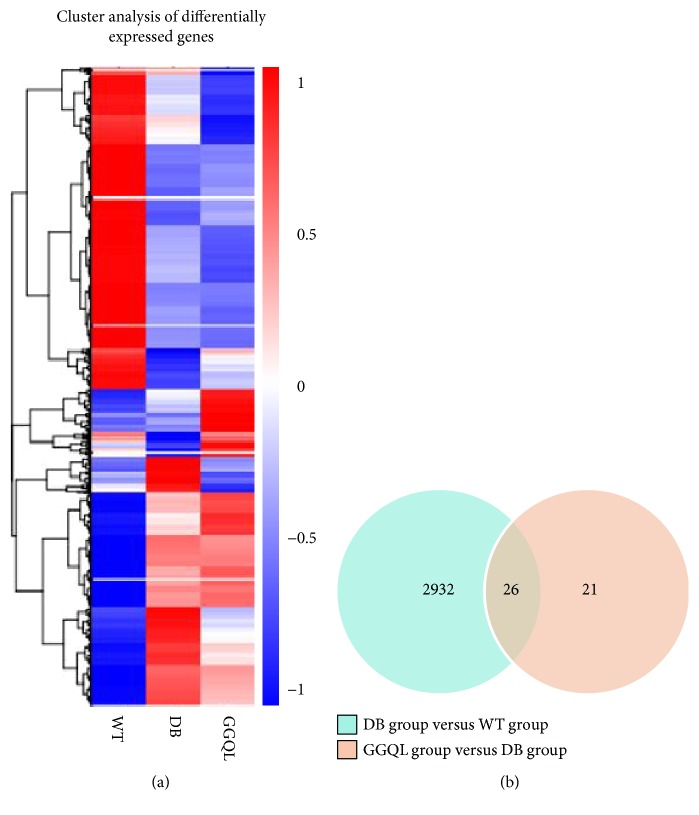
(a) Heatmap for cluster analysis of differentially expressed genes. (b) Venn diagram of differentially expressed genes.

15 samples from the WT group, DB group, and GGQL group were detected for DEGs, with 5 samples in each group. The hierarchal clustering results showed that 2958 genes were detected to be differentially expressed when comparing the DB group with the WT group, including 1450 upregulated genes and 1508 downregulated genes. Compared to the DB group, 47 genes were differentially regulated in the GGQL group (Figure 3). Among them, 26 genes were also differentially regulated in the DB group versus the WT group (Table 2), and 21 genes expressed no difference in the DB group versus the WT group (Table 3). The genes differentially expressed were related to anion binding, oxidoreductase activity, calcium-dependent phospholipid binding, ceramide metabolic process, and others.

### 3.4. Validation of Differentially Expressed Genes

Four differentially expressed genes were selected to validate by RT-qPCR, including Acer2, Slc38a2, Ppp1r3c, and Pgam1. Acer2 (alkaline ceramidase 2) regulates the hydrolysis of ceramides. Slc38a2 (solute carrier family 38 member 2) is a sodium-dependent amino acid transporter. Ppp1r3c (protein phosphatase 1 regulatory subunit 3C) is a glycogen targeting subunit of PP1 and regulates glycogen metabolism. Pgam1 (phosphoglycerate mutase 1) is an enzyme which catalyzes the reaction of 3-phosphoglycerate (3-PGA) to 2-phosphoglycerate (2-PGA).

**Figure 4:**
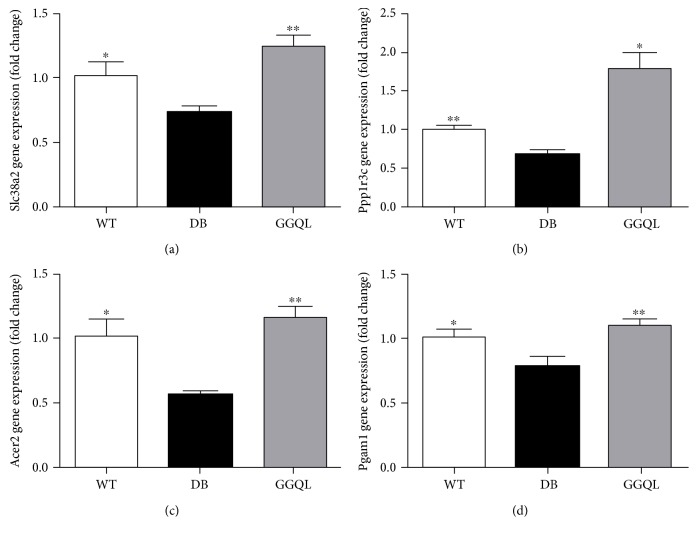
The results of RT-qPCR to validate differentially expressed genes. The mRNA expressions of (a) Slc38a2, (b) Ppp1r3c, (c) Acer2, and (d) Pgam1 in each group (*n* = 4 − 5). The DB group was the reference group to calculate *P* values, ^∗^*P* < 0.05 and ^∗∗^*P* < 0.01.

The results of RNA-seq showed that, compared with the WT group, the expressions of Acer2, Slc38a2, Ppp1r3c, and Pgam1 were depressed in LV tissue of the DB group mice. And the expressions of all the four genes were significantly upregulated in the GGQL group. The results of RT-qPCR were consistent with the results of RNA-seq, and the results of RNA-seq were validated (Figure 4).

### 3.5. Content of Ceramide in Myocardial Tissue

**Figure 5:**
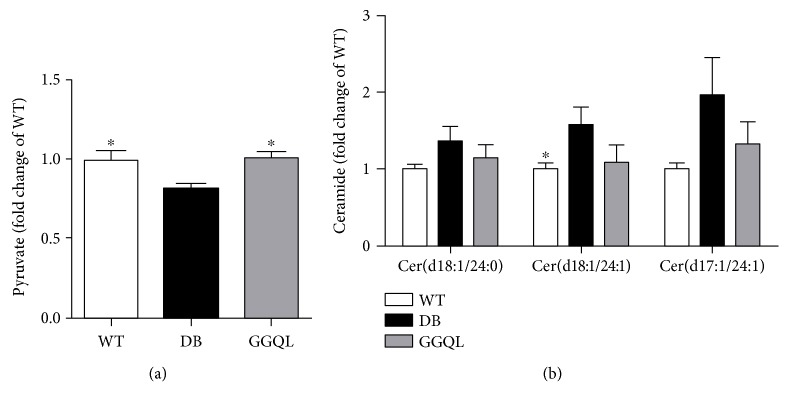
The results of pyruvate and ceramide content in the myocardial tissue. The content of (a) pyruvate and (b) ceramide in each group (*n* = 3–6). The DB group was the reference group to calculate *P* values, ^∗^*P* < 0.05.

The content of ceramide (d18:1/24 : 1) in the myocardial tissue of the model group was markedly higher than that of the wild-type group (*P* < 0.05). In the model group, the content of ceramide (d18:1/24 : 0) and ceramide (d17:1/24 : 1) appeared an increasing trend compared to that of the wild-type group. The levels of ceramide (d18:1/24 : 1), ceramide (d18:1/24 : 0), and ceramide (d17:1/24 : 1) in the myocardial tissue of the GGQL group had a reduced trend compared to that of the model group (Figure 5).

### 3.6. Content of Pyruvate in Myocardial Tissue

In the model group, the pyruvate content of myocardial tissue homogenate decreased significantly than that of the wild-type group (*P* < 0.05). Compared to the model group, the content of pyruvate in the myocardial tissue homogenate of the GGQL group increased obviously (*P* < 0.05) (Figure 5).

## 4. Discussion

DM is not only manifested as long-term and chronic elevated hyperglycemia but also accompanied with various complications. Diabetic cardiomyopathy (DCM) is one of the major causes of death in T2DM patients. DCM is a type of primary cardiomyopathy, independent of macrovascular and coronary artery diseases. The pathophysiology of DCM includes cardiomyocyte hypertrophy, focal necrosis, extracellular matrix accumulation, and interstitial fibrosis [12]. Early manifestations usually expressed as diastolic dysfunction with major pathological changes of declining in myocardial compliance and blocked diastolic filling [13]. Systolic dysfunction is the major pathological change of DCM in the late period of the disease, and this disturbance is prone to congestive heart failure.

db/db mice are the classic models for the research of T2DM-induced DCM. A number of investigators confirmed the existence of DCM of db/db mice with evaluation of echocardiography [14, 15]. According to the literature, under the detection of echocardiography, the *E*/*A* ratio and *E*'/*A*' ratio of the db/db mice decreased significantly compared with WT mice [14, 16, 17], suggesting impaired cardiac diastolic function.

In our study, compared with the WT mice, the *A* wave increased, and the left ventricular *E*/*A* and *E*'/*A*' ratio of the db/db mice markedly decreased, suggesting a diastolic dysfunction of the left ventricular; Gegen Qinlian decoction could significantly increase the left ventricle *E*/*A* and *E*'/*A*' ratio of the db/db mice and downregulate the *A* wave, suggesting that the drug can improve the function of the left ventricle.

Glycolysis is a process in which one molecule of glucose is converted to two molecules of pyruvate and produces two molecules of adenosine triphosphate (ATP), which provides energy for almost all the biological processes. Glycolysis is necessary for cardiac metabolism. Glucose utilization is impaired, meanwhile glycolysis and glucose oxidation are depressed in DCM. In diabetes, the capacity of cardiac glycolysis is chronically reduced [18]. The reduction of myocardial glycolysis may be associated with the development of heart failure and myocardial injury suffered by DM patients [19, 20].

Glycolysis consists of ten steps, and pyruvate is the product. Phosphoglycerate mutase-1 (Pgam1) is used to catalyze the interconversion of 3-phosphoglycerate (3-PGA) to 2-phosphoglycerate (2-PGA), which is the eighth step of glycolysis [21].

Ceramide is a main molecule of the sphingolipid metabolism, which mediates cell growth retardation and differentiation, and inhibits cell proliferation and promotes apoptosis [22–26]. The accumulation of tissue ceramides may be helpful to the development of insulin resistance [27]. Ceramide is an important mediator of myocardial lipid toxicity [28, 29]. Moreover, some studies showed that ceramide is related to cardiomyocyte apoptosis induced by ischemia/reperfusion injury [30, 31]. Alkaline ceramidase 2 (Acer2) is a type of ceramidases, which are key enzymes of the degradation of intracellular ceramide and hydrolyzed ceramide to sphingosine and then further phosphorylated to sphingosine-1-phosphate (S1P) by sphingosine kinase [32]. And the increased expression of Acer2 may cause the decrease in ceramide accumulation.

Previous research indicated that the cardiac glycolytic rate of db/db mice was reduced. Cardiac function, glycolysis, and glucose oxidation of isolated hearts of C57BL/KsJ (*db*/*db*) were damaged [19]. It is also reported that in the diabetic model animals, myocardial ceramide content was significantly increased accompanied with cardiac dysfunction [33, 34].

The results of this study showed that compared with the mice of the WT group, the mRNA content of Pgam1 and Acer2 in myocardium of the DB group mice decreased significantly. And the mRNA content of Pgam1 and Acer2 in myocardium of the GGQL group mice increased significantly, compared with the mice of the DB group. It indicated that the myocardial glycolysis and ceramide hydrolysis were depressed in the DB group mice, and potentially the treatment of Gegen Qinlian decoction protects the myocardium by promoting glycolysis and decreasing the content of ceramide. In this study, the results of the pyruvate content in the myocardial tissue suggested that the glycolysis decreased in the DB group compared to the WT group. And GGQL decoction has the effect to increase cardiac glycolysis in diabetic mice. The content of ceramide in the myocardial tissue of the DB group appeared an increasing trend compared to that of the wild-type group, and GGQL decoction trended to reduce the ceramide content in diabetic mice. The results were consistent with the results of RNA-seq and q-PCR.

Previous studies have shown that berberine has protective effect on the development of diabetic cardiomyopathy [35]. Liquiritigenin and liquiritin have a protective role in high fructose-induced myocardial fibrosis [36, 37]. Other investigators confirmed that various components of Gegen Qinlian decoction have moderating effect on glycolysis and ceramide levels. Berberine could significantly reduce serum levels of ceramide of nonalcoholic fatty liver disease patients [38] and stimulate the glycolysis in HepG2 hepatocytes and C2C12 myotubes [39]. Berberine compounds could upregulate the glycolysis which was depressed in hyperlipidemic rats [40]. Baicalin could decreased the blood glucose of diabetic rats by increasing the content of liver glycogen and promoting glycolysis [41]. It is concluded that the effect on improving diastolic function of the left ventricle and promoting the mRNA expression of Pgam1 and Acer2 of Gegen Qinlian decoction may be attributed to berberine, baicalin, liquiritigenin, and liquiritin.

In conclusion, the results of this study showed that Gegen Qinlian decoction has a therapeutic effect on diastolic dysfunction of the left ventricle in db/db mice, and the effect may be related to its role in promoting myocardial glycolysis and decreasing the content of ceramide.

## Figures and Tables

**Table 1:** List of primers used in RT-qPCR.

Gene	Primer sequence (5′–3′)
Pgam1	F:AATTCAGGGAGGAACTGTGCT R:GGACAGGTTCCAGGGACAAAA

Acer2	F:GCTCTGTGAAAATACTGCCACC R:CAGTGTTGGCTCTGGGTAGG

Slc38a2	F:CAAACCTCCTGTGAGGGAGC R:GAATTGAGGTGACGGGACAGT

Ppp1r3c	F:AGAGCTCTTTCAGTGCCTCCA R:TGATGGCCCTCCTGATGATTTC

F: forward; R: reverse.

**Table 2:** Genes significantly regulated in the GGQL group versus the DB group and also significantly regulated in the DB group versus the WT group.

Gene name	Description	Adjusted *P* value (DB/WT)	Adjusted *P* value (GGQL/DB)
Pgam1	Phosphoglycerate mutase 1	<0.0001	0.0038
Igf1	Insulin-like growth factor 1	0.0060	0.0029
Cpeb4	Cytoplasmic polyadenylation element binding protein 4	0.0141	0.0283
Slc38a2	Solute carrier family 38, member 2	0.0000	<0.0001
Vegfa	Vascular endothelial growth factor A	0.0001	0.0005
Banp	BTG3-associated nuclear protein	<0.0001	0.0100
Rbm38	RNA-binding motif protein 38	0.0078	0.0380
Rasgef1b	RasGEF domain family, member 1B	0.0003	0.0037
Anxa3	Annexin A3	<0.0001	0.0309
Cyp1a1	Cytochrome P450, family 1, subfamily a, polypeptide 1	<0.0001	0.0016
Trib1	Tribbles homolog 1 (Drosophila)	0.0004	0.0118
Nphp3	Nephronophthisis 3 (adolescent)	<0.0001	0.0275
Fhl3	Four and a half LIM domains 3	0.0036	0.0045
Serpine1	Serine (or cysteine) peptidase inhibitor, clade E, member 1	<0.0001	0.0016
Acer2	Alkaline ceramidase 2	<0.0001	0.0016
Arhgap32	Rho GTPase activating protein 32	<0.0001	<0.0001
Atf4	Activating transcription factor 4	0.0044	0.0016
Zc3h6	Zinc finger CCCH type containing 6	<0.0001	0.0136
Tet1	Tet methylcytosine dioxygenase 1	0.0006	0.0016
Klk1b26	Kallikrein 1-related petidase b26	0.0146	0.0189
Ppp1r3c	Protein phosphatase 1, regulatory (inhibitor) subunit 3C	0.0000	0.0029
Ttc30b	Tetratricopeptide repeat domain 30B	<0.0001	0.0054
Fign	Fidgetin	<0.0001	0.0195
AI593442	Expressed sequence AI593442	<0.0001	0.0350
5730480H06Rik	RIKEN cDNA 5730480H06 gene	0.0019	0.0029
Gm26703	Predicted gene, 26703	<0.0001	<0.0001

Genes with an adjusted *P* value < 0.05 were considered to be differentially expressed.

**Table 3:** Genes significantly regulated in the GGQL group versus the DB group but no differential expression in the DB group versus the WT group.

Gene name	Description	Adjusted *P* value (DB/WT)	Adjusted *P* value (GGQL/DB)
Gatsl3	GATS protein-like 3	0.4265	<0.0001
Abca8b	ATP-binding cassette, subfamily A (ABC1), member 8b	1	0.0195
Apex2	Apurinic/apyrimidinic endonuclease 2	0.8103	0.0077
Dnajb4	DnaJ (Hsp40) homolog, subfamily B, member 4	0.1556	0.0071
Vgll4	Vestigial-like 4 (Drosophila)	0.9649	0.0136
Klhl36	Kelch-like 36	0.1950	0.0136
Amotl2	Angiomotin-like 2	0.0637	0.0030
Cish	Cytokine inducible SH2-containing protein	0.4114	<0.0001
Myo6	Myosin VI	1	0.0483
Caskin2	CASK-interacting protein 2	0.8910	0.0158
Baz1a	Bromodomain adjacent to zinc finger domain 1A	0.2177	0.0020
Fbxw17	F-box and WD-40 domain protein 17	0.6014	0.0480
Ddx60	DEAD (Asp-Glu-Ala-Asp) box polypeptide 60	0.9151	0.0097
Gck	Glucokinase	0.1227	0.0112
Zfp507	Zinc finger protein 507	0.6355	0.0201
Zfp518a	Zinc finger protein 518A	1	0.0416
Dusp7	Dual specificity phosphatase 7	0.7537	0.0416
Ifnlr1	Interferon lambda receptor 1	0.3676	0.0275
Rnasel	Ribonuclease L (2′,5′-oligoisoadenylate synthetase-dependent)	1	0.0275
Zfp763	Zinc finger protein 763	0.2005	0.0063
RP23-402K24.5	RIKEN cDNA 6230415J03 gene	0.4670	0.0350

Genes with an adjusted *P* value < 0.05 were considered to be differentially expressed.
